# The Feasibility of Telerobotic Pancreaticojejunostomy Using a Surgical Robot: A Pilot Study With Commercial Optical Networks

**DOI:** 10.1111/ases.70181

**Published:** 2025-11-23

**Authors:** Yusuke Wakasa, Kenichi Hakamada, Hajime Morohashi, Kazuki Yokoyama, Yuma Ebihara, Satoshi Hirano, Eiji Oki, Norihiko Ikeda, Akinobu Taketomi, Masaki Mori

**Affiliations:** ^1^ Department of Gastroenterological Surgery Hirosaki University Graduate School of Medicine Hirosaki Japan; ^2^ Committee for Promotion of Remote Surgery Implementation Japan Surgical Society Tokyo Japan; ^3^ Department of Gastroenterological Surgery II, Faculty of Medicine, Graduate School of Medicine Hokkaido University Sapporo Japan; ^4^ Center for Integration of Advanced Medicine, Life Science and Innovative Technology (CAMIT) Kyushu University Hospital Fukuoka Japan; ^5^ Department of Surgery Tokyo Medical University Tokyo Japan; ^6^ Department of Gastroenterological Surgery I Hokkaido University Graduate School of Medicine Sapporo Japan; ^7^ Tokai University School of Medicine Tokyo Japan

**Keywords:** pancreaticojejunostomy, telesurgery, telesurgical guidance

## Abstract

**Introduction:**

In recent years, the practical application of remote robotic surgery has become a reality, and is expected to be applied to difficult surgeries. The purpose of this study is to demonstrate whether the pancreaticojejunostomy in a pancreaticoduodenectomy, a difficult surgery, can be performed through a remote surgery‐assisted robotic operation, as well as to verify the feasibility of remote surgery‐assisted pancreaticojejunostomy.

**Methods:**

Hirosaki city and Goshogawara city (about 30 km) were connected via a commercial communication line using the hinotori surgical robot, and five surgeons performed remote surgery on an artificial organ model for pancreaticojejunostomy. Four local surgeons were instructed remotely. Each procedure was repeated 3–5 times in sets of 8 min, and communication latency, Image Quality Score, System Usability Scale (mSUS), and Robot Usability Score were all evaluated.

**Results:**

The communication latency was stable at less than 12 msec, and there were no problems in performing the surgery. No significant differences were noted in Image Quality Score, System Usability Scale (mSUS), or Robot Usability Score. Pancreaticojejunostomy was performed using the Blumgart anastomosis technique, and all procedures were completed without any issues.

**Conclusion:**

We demonstrated that pancreaticojejunostomy can be performed in a telesurgical environment. This system can be applied to remote surgical guidance and support in the future and it is expected to correct regional disparities in medical care, improve surgical education, and enhance the implementation of remote surgery in society.

## 1 Introduction

In recent years, advances in surgical robotics technology, coupled with the development of high‐speed, high‐capacity communication technology, have raised expectations for the practical application of telerobotic surgical systems based on these technologies. In addition, there is growing interest in the role of telesurgery as a tool for solving social problems such as rural medical disparities and surgeon shortages [1, 2, 3]. Our research group has conducted about 20 demonstration studies in Hokkaido [4], Aomori Prefecture [5, 6, 7, 8, 9], and the Kyushu region [10] toward the construction and social implementation of a robotic telesurgery system and formulated Japanese telesurgery guidelines based on these results [11]. In principle, the Japanese online practice guidelines apply to D to P with D (Doctor to Patient with doctor) cases where there is a surgeon on site, and full telesurgery is not permitted. Therefore, telesurgical support to educate young surgeons is considered to be one of the most useful applications of robotic telesurgery.

In previous empirical studies, robotic telesurgery and telesurgical guidance in relatively low‐difficulty procedures such as intestinal anastomosis and cholecystectomy have been performed [8, 9, 12, 13]. Conversely, the application of robotic telesurgery and telesurgical guidance has not been sufficiently investigated for difficult procedures that are assumed to require much surgical assistance from a senior surgeon in the real world. In the field of gastrointestinal surgery, most intestinal anastomoses are performed by instrumentation, and in the era of robotic surgery, the opportunities for intracorporeal instrumentation are increasing [14]. Unlike intestinal anastomosis, pancreaticojejunostomy for pancreaticoduodenectomy (PD) is unsuitable for instrumented anastomosis due to the fine anastomotic sutures, so nodal sutures are mainly used. The 3D stereoscopic view, magnification effect, and anti‐shaking function of robotic surgery have the potential to realize sutures with an accurate needle movement in pancreaticojejunostomy. However, stabilization of the technique for remote pancreaticojejunostomy will require a great deal of experience and support from skilled physicians. Therefore, it is highly significant to verify the extent to which remote surgical support can be provided to distant areas in order to offer advanced medical care in locations with few specialists. The purpose of this study was to verify the feasibility and safety of robotic remote surgery and remote surgical guidance for PD, one of the most difficult surgeries, and to obtain basic knowledge for the future development of this complicated surgery.

## 2 Materials and Methods

### 2.1 Network Connections

**FIGURE 1:**
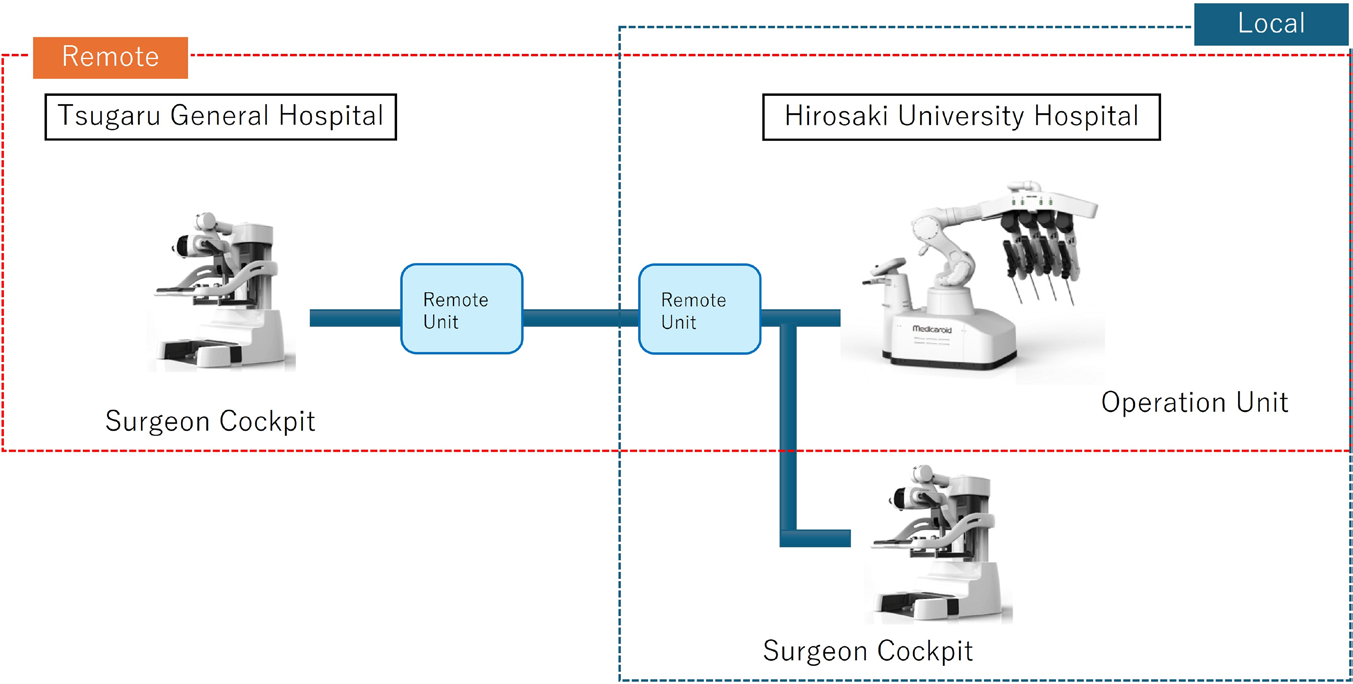
Network system: Hirosaki University Hospital and Tsugaru General Hospital, 30 km apart, were connected by a fiber optic network provided by Nippon Telegraph and Telephone East Corporation.

Hirosaki University Hospital (Hirosaki City, Aomori Prefecture) and Tsugaru General Hospital (Goshogawara City, Aomori Prefecture), located about 30 km north of Hirosaki City, were connected using commercial optical fiber lines. The optical fiber network used communication lines provided by NTT East Corporation (Tokyo, Japan). Three types of lines were provided: a guaranteed type line (maximum speed 100 Mbps), a best‐effort type line (maximum speed 1 Gbps), and an Innovative and Optical Wireless Network (IOWN), that is, a next‐generation high‐capacity line (All‐Photonics Connect powered by IOWN). Communication information was compressed and decompressed using a Medicaroid remote unit (Figure 1).

### 2.2 Surgical Robot System

The robot used was hinotori, a robotic surgical system developed by Medicaroid Corporation (Tokyo, Japan) [15]. The cockpit was installed at Hirosaki University Hospital and the surgeon's console at Tsugaru General Hospital. Technical support, including the installation of encoders and decoders for establishing the communication environment, was provided by Medicaroid Corporation.

### 2.3 Telesurgery and Telesurgical Guidance

**FIGURE 2:**
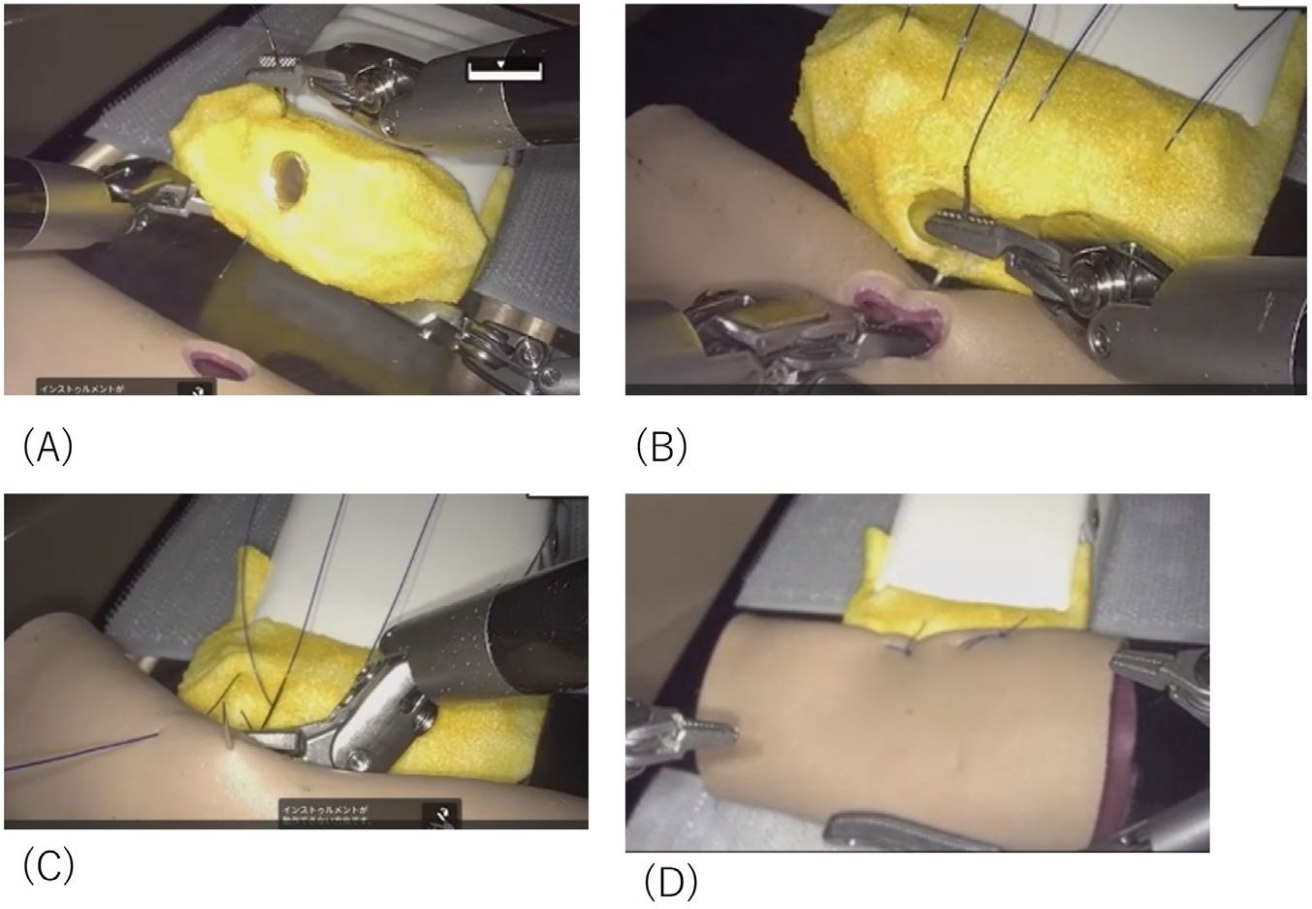
Artificial organ model for Pancreaticojejunostomy. (A) Pancreatic parenchyma‐to‐seromuscular jejunal anastomosis (dorsal side) (B) Duct‐to‐mucosa pancreaticojejunostomy (C) Pancreatic parenchyma‐to‐seromuscular jejunal anastomosis (ventral side) (D) Anastomosis completed.

Telesurgery was performed on pancreaticojejunostomy organ models (Figure 2) [16] under remote conditions by five hepatopancreatobiliary (HPB) surgeons, each of whom had prior experience with more than 10 laparoscopic or robot‐assisted pancreatic resections. Subsequently, four surgeons without subspecialty training in HPB surgery and without prior experience in robot‐assisted pancreatic resections participated as local operators, and telesurgical guidance was provided to them by four of the previously mentioned HPB surgeons. The duration of the surgery was 3–5 sets of 8 min each. All telesurgeries and local surgeons were asked to complete questionnaires to evaluate various operability levels: Image Quality Score, mSUS, and Robot Usability Score, which assess the type of image quality degradation caused by changes in the communication environment and effects on the procedure. This process includes scores for clarity, stereopsis, completeness, continuity, and impact on technique, with higher scores on a 5‐point scale indicating no degradation in image quality and no impact on the procedure (Table 1). The total score for each item was used in the evaluation. The mSUS was a modified version of the System Usability Scale (SUS) proposed by Brooke [17]. Nine items were rated on a 5‐point scale, and total scores were calculated. Again, a higher score indicates a higher evaluation, while a lower score indicates a lower assessment (Table 2). The effectiveness criteria, which we developed to evaluate eight items, are shown in Table 3. Robot operability was rated on a 5‐point scale in order of ease of operation. Subjective feedback and assessments of perceived learning effects were collected from the local surgeons in free‐text format.

**TABLE 1:** Image quality score (IQS).

Please answer the following questions about the surgical images of the robotic surgery you performed				
On a scale of 1 to 5, with 5 being considered satisfactory for performing the procedure and 0 being considered completely unsuitable for performing the procedure, please rate the procedure				
Clarity:How clear were the images from the robotic surgery?				
It's not clear at all		Very clear		
1	2	3	4	5
2 Stereoscopic vision:Were the images of the robotic surgery in stereoscopic vision?				
Was not stereoscopic vision at all		Very stereoscopic vision		
1	2	3	4	5
3 Completeness:Was the robotic surgery screen complete?				
Incomplete		Complete		
1	2	3	4	5
4 Continuity:Were the robotic surgery screens continuous?				
There's no continuity at all		It's completely continuous		
1	2	3	4	5
5 Impact on the procedure:Were you able to perform the procedure with the images from this robotic surgery?				
Could not perform at all		Could be done perfectly		
1	2	3	4	5

*Note:* Total score: The degradation of image quality was evaluated using a five‐step evaluation scale.

**TABLE 2:** Modified System usability scale (mSUS).

Please answer the following questions about the remote robotic surgical environment				
1: I don't think so at all	2: I don't think so	3: Neither	4: I think so	5: I strongly think so
1. I think I'll make sure I use this system often				
1	2	3	4	5
2. This system is simple and easy to use				
1	2	3	4	5
3. This system is easy to use				
1	2	3	4	5
4. I don't need technical support personnel to use this system				
1	2	3	4	5
5. Many people will be able to use this system very soon				
1	2	3	4	5
6. Gave me the confidence to use this system				
1	2	3	4	5
7. This system is intuitive and easy to us				
1	2	3	4	5
8. I found the functionality of this system very useful				
1	2	3	4	5
9. This system was a great help in carrying out this task				
1	2	3	4	5

**TABLE 3:** Robot usability score.

Please answer the following questions about the tele‐robotic surgery environment				
1: I don't think so at all	2: I don't think so	3: Neither	4: I think so	5: I strongly think so
1. I was physically comfortable				
1	2	3	4	5
2. I had good hand control in this environment				
1	2	3	4	5
3. I had good foot control in this environment				
1	2	3	4	5
4. I had a good 3D field of view in this environment				
1	2	3	4	5
5. I had no complaints and felt little stress in this environment				
1	2	3	4	5
6. The robot moved smoothly				
1	2	3	4	5
7. The robot did exactly what I wanted it to do				
1	2	3	4	5
8. I think I can actually perform surgery using this type of robotic surgery environment				
1	2	3	4	5
9. If you answered 1–3 to question 8, please choose the reason from the following. If you have any other reason, please provide it as a free answer. The image was rough The robot didn't work the way I wanted it to My technique was inexperienced Other()				

*Note:* Name:  Task No.  1 · 2 Total score:  /40.

### 2.4 Surgical Techniques for the Artificial Organ Model

Pancreaticojejunostomy was performed using the modified Blumgart method, which is routinely employed at our institution. In the pancreaticojejunostomy model, the anastomosis is sutured through the pancreatic parenchyma from the ventral side without suturing the main pancreatic duct using a 4–0 polypropylene suture (15 cm) 1/8 circumferential double‐ended needle with a non‐absorbable thread, followed by suturing dorsally through the small hole in the jejunum and again dorsally through the parenchyma. One suture is placed on the head side of the main pancreatic duct and one on the caudal side (Figure 2 (A)). Next, for the pancreatic duct jejunal mucosa anastomosis, 5–0 polydioxanone sutures are cut into 10 cm lengths and ligated with 3 stitches on the pancreatic duct side and the intestinal side, respectively, using a 1/2 circumferential needle for the pancreatic duct posterior wall and a 3/8 circumferential needle for the pancreatic duct anterior wall (Figure 2B). Finally, 4–0 polypropylene sutures are placed and ligated to the pancreatic parenchyma and jejunal muscularis mucosae respectively, to cover the pancreatic mucosa anastomosis, and the anastomosis is completed by suture ligation of the ventral jejunal muscularis mucosae head side and tail side (Figure 2C,D).

### 2.5 Statistical Analysis

Statistical analyses were performed using IBM SPSS Statistics for Windows, version 29.0 (IBM Corp., Armonk, NY, USA). For Image Quality Score, mSUS, and Robot Usability Score, comparisons among the three groups—telesurgery, telesurgical guidance, and local operation—were conducted using the Kruskal–Wallis test, and *p* values were calculated.

## 3 Results

### 3.1 Telesurgery

In all five cases, surgeons were able to perform pancreaticojejunostomy as a telesurgery without encountering problems. The mean Image Quality Score was 22.8 (21–25), the mean mSUS was 28.8 (23–35), and the mean Robot Usability Score was 30.5 (28–33). (Table 4).

**TABLE 4:** IQS, mSUS, robot usability score for the experiment. Values are averages (ranges).

	Telesurgery	Telesurgical support	Local	χ^2^	*p*
Image quality score (Max 25)	22.8 (21–25)	23.0 (21–25)	24.3 (22–25)	2.60	0.305
mSUS (Max 45)	30.8 (23–39)	30.5 (19–39)	33.2 (29–38)	0.21	0.888
Robot usability score (Max 40)	32.4 (28–40)	30 (22–35)	35.2 (31–40)	1.09	0.579

### 3.2 Telesurgical Guidance

The tele‐assistant in all four pairs was able to provide telesurgical guidance for pancreaticojejunostomy; however, one of the local surgeons was unable to complete the procedure due to time constraints. The teaching tool, annotation, could be used without issues, and swapping between operators could be performed without any problems. The mean Image Quality Score, mSUS, and Robot Usability Score for the tele‐assistants came to 23.0 (21–25), 30.5 (19–39), and 30.0 (22–35), respectively. For local surgeons, the mean Image Quality Score was 24.3 (22–25), the mean mSUS was 33.2 (29–38), and the mean Robot Usability Score was 35.2 (31–40). There were no significant differences in Image Quality Score, mSUS, or Robot Usability Score for telesurgery, telesurgical guidance among local surgeons (Table 4).

### 3.3 Communication Delay

The average communication latency was 12 (10–14) msec for guaranteed lines at 100 Mbps, 11 (10–17) msec for best‐effort lines at 1 Gbps, and 2 (1–7) msec for IOWN lines at 10 Gbps (Table 5). The bandwidth used from 15 to 25 Mbps, excluding the video compression bandwidth.

**TABLE 5:** Delay time for each line.

Line	Mean [min.‐max.] (msec)
Guaranteed 100Mbps	12 [10–14]
Best effort 1G	11 [10–17]
IOWN 1Gbps	2 [1–7]

## 4 Discussion

In this study, both telesurgery and telesurgical guidance for the pancreaticojejunostomy model were successfully performed. The maximum communication delay was 12 msec, which was acceptable and did not affect the operation. There were no problems with surgeon operability.

PD remains one of the most difficult procedures in gastrointestinal surgery, with a complication rate of 30%–50% [18, 19, 20, 21] and a mortality rate of approximately 5% [20, 22, 23]. Postoperative pancreatic fistula (POPF) is one of the most common complications, and severe cases are classified as grade B/C (Clinically Relevant: CR‐POPF) by the International Study Group on Pancreatic Surgery (ISGPS). There are many risk factors for the development of POPF, but the skill of the surgeon is the most important factor; therefore, improvement in surgical technique is of paramount importance. Minimally invasive surgery in PD has progressed these days, and laparoscopic and robot‐assisted surgery have become widespread [24]. Recently, robot‐assisted PD has been reported to improve the R0 resection rate, decrease blood loss, and shorten hospital stay. Although the operation time tends to be longer than that of open PD [25], there is an increasing demand for the training and improvement of surgeons in robot‐assisted surgery as well as open surgery.

In the past 10 years, reports on learning curves in robot‐assisted surgery have been published. While some authors have reported that it takes approximately 40–90 cases to complete the initial phase of mastering laparoscopic surgery [26, 27, 28, 29, 30], the learning curve for robotic surgery is actually shorter than that of laparoscopic surgery, from 25 to 44 cases [31, 32, 33]. The reasons for this are thought to be that robot‐assisted surgery has technical advantages over laparoscopic surgery, such as high‐quality 3D images, freely moving articulated forceps, and image stabilization, which may shorten the learning curve compared to laparoscopic surgery [31]. It is also speculated that one of the factors contributing to this is the availability of enhanced educational tools in robotic surgery, such as the annotation and swapping functions. Therefore, robot‐assisted surgery can be evaluated as having excellent educational characteristics for surgeons that are not present in open or laparoscopic surgery. Furthermore, with regard to the learning curve for pancreaticoduodenectomy (PD), a systematic review comparing open, laparoscopic, and robotic approaches has been reported. Muller et al. demonstrated that more than 100 cases are required for stabilization in open PD, 40–60 cases for laparoscopic PD, and 20–40 cases for robotic PD, and they emphasized the need to standardize the definition and evaluation of learning curves [34]. These findings support the educational advantage of robot‐assisted surgery demonstrated in the present study and suggest that robotic technology may facilitate earlier proficiency even in highly complex procedures such as PD.

It has been regarded as challenging to perform highly difficult surgeries such as PD in a remote environment because of the possibility of such procedures being negatively affected by communication delays. This is because communication delay issues, which are the biggest hurdle in remote surgery, have yet to be addressed and ameliorated. The most significant cause of these delays is considered to be the transmission of images containing a large amount of information. Delay occurs when the image information is compressed and restored by the information processing technology of the encoder/decoder and transmitted over the communication line. In our previous report on delays in telerobotic surgery and our previous demonstration experiments, we mentioned that delays exceeding 100 msec may affect the surgical procedure and may be a criterion that makes it difficult to proceed with the surgery [13, 35, 36]. In addition, Nakauchi et al. have proposed 125 ms as a practical threshold for latency in telesurgery [37]. However, the maximum communication delay in the present study was less than 12 msec, and it was confirmed that the communication delay was not a problem regardless of whether a guaranteed, best‐effort, or IOWN line was used, suggesting that the technology to control communication and video delay has reached a sufficiently practical level.

In this study, the operability and image quality evaluation questionnaires for telesurgery, telesurgical guidance, and local surgeon impressions indicated that surgery could proceed without significant stress or restriction to the surgeon, regardless of whether the surgery was performed remotely, with telesurgical guidance, or at a remote or local site. This, presumably, is due to the technical advantages of the current surgical robot, such as its precise operation support mechanism, multi‐degree‐of‐freedom forceps structure, and high three‐dimensional visibility of the surgical field, as described above, as well as the improvement of communication delay control technology, also delineated above. The development of telesurgical guidance using robotic telesurgery technology will not only make it possible to perform high‐difficulty surgical support, such as for PD, in remote areas in the near future, but also to establish a system in which specialists at the core hospital can provide real‐time guidance and intervention, thereby enabling the proliferation of remote procedures. In addition, establishing a system in which specialists at core hospitals can provide real‐time guidance and intervention may contribute to the enhancement of the education of local surgeons on difficult surgeries, and ultimately to improve robotic surgical techniques in Japan as a whole.

Still, however, there are some issues to be overcome before this technology can be applied to actual clinical practice. First, the artificial organ models and simulated environment used in this study do not fully replicate the complexities of actual clinical practice, such as tissue fragility, bleeding, inflammatory responses, and unexpected intraoperative complications. Therefore, the present findings cannot be directly applied to clinical practice, and further validation is required to bridge this gap. Specifically, safety and efficacy should first be assessed in animal models, followed by carefully designed clinical trials involving selected cases with appropriate ethical considerations. Establishing such a roadmap will be essential to ensure that this technology is introduced into clinical practice in a manner that maximizes patient benefit. In addition, it is essential to be prepared for unexpected events such as intraoperative bleeding and communication failures. It is important to prepare for such events, at preoperative conferences, by establishing criteria for changing to other surgical procedures and by pre‐determining personnel assignments for emergency rollout. Furthermore, standardization of redundant configurations in case of communication failures and the dissemination of staff education materials and technical training at actual facilities should also be promoted under institutional support. In the future, safety, effectiveness, and treatment outcomes of telesurgery should be evaluated from multiple perspectives through clinical studies on actual patients.

In conclusion, this study provides a new perspective on the clinical application of telemedicine in that it demonstrates that robotic telesurgery is safe and technically feasible for pancreaticojejunostomy, one of the most advanced procedures in the field of gastrointestinal surgery. In order to promote the social implementation of robotic telesurgery, it is important to further strengthen the technological infrastructure, including communication and operation technologies. It is also necessary to develop an institutional framework and accumulate clinical evidence through multicenter collaborative research.

## 5 Limitations

This study has several limitations. First, the verification time was limited, resulting in a small number of participants, and tasks could not be performed in a randomized fashion. Furthermore, the study was restricted to the use of limited wired communication lines. Second, the inter‐institutional distance was approximately 30 km, a relatively short distance within driving range, which represents another limitation. Although this study was designed with telesurgical guidance in underserved areas in mind, validation in longer‐distance settings with greater geographic constraints is necessary. Third, this study utilized dry organ models; therefore, additional validation in animal experiments and actual clinical environments is required. In addition, objective evaluations of anastomotic safety, such as leak testing or histological assessment, were not performed, and this remains a limitation and an important future task. Fourth, the present study focused exclusively on the reconstructive portion (pancreaticojejunostomy) of PD and did not assess the resection phase. Because resection carries a significant risk of bleeding, which could have a substantial impact on the safety of telesurgery and telesurgical guidance, experimental and clinical validation that includes the resection phase will be necessary. Finally, regarding audio transmission, voice communication was conducted using the same commercial optical line and the built‐in voice communication tool of the hinotori system. However, in this system it was not possible to separately evaluate audio and video transmission, and objective assessment of audio latency could not be performed in this study. Moreover, operative time was not measured or analyzed, and the absence of this fundamental parameter should also be recognized as a limitation.

## Author Contributions

Yusuke Wakasa designed the study, analyzed the data, interpreted the results, and wrote the manuscript. Kenichi Hakamada provided overall study supervision and guidance, as well as interpretation and analysis of results. Hajime Morohashi validated the study design, was responsible for data collection, interpreted the results, and provided analysis. Kazuki Yokoyama contributed to the data collection and analysis. Yuma Ebihara, Satoshi Hirano, Eiji Oki, Norihiko Ikeda, Akinobu Taketomi, and Masaki Mori supervised this study. All authors reviewed the final manuscript.

## Ethics Statement

This study does not fall into the category of “life science/medical research involving human subjects” and thus does not need to address or provide an ethical application. Surgeons used robotic technology to operate on artificial (non‐living) organs in a simulated telesurgical situation. Formal opt‐out was obtained from the Hirosaki University Ethics Committee for this study, which included the above details (approval number: 2022–081).

## Consent

All subjects (participating surgeons) were notified in writing about the study and gave written consent.

## Conflicts of Interest

Yusuke Wakasa, Kazuki Yokoyama, and Masaki Mori declare that they have no conflicts of interest to disclose. Kenichi Hakamada, Hajime Morohashi, Yuma Ebihara, Satoshi Hirano, Eiji Oki, Norihiko Ikeda, and Akinobu Taketomi received research support from the Japan Agency for Medical Research and Development (AMED) under Grant Number 21hs0122001h0002. All other authors have no relevant financial or non‐financial interests to disclose.

## Data Availability

Data sharing not applicable to this article as no datasets were generated or analysed during the current study.
